# Functional Evaluation and Genetic Landscape of Children and Young Adults Referred for Assessment of Bronchiectasis

**DOI:** 10.3389/fgene.2022.933381

**Published:** 2022-08-08

**Authors:** Jeffrey Fong Ting Chau, Mianne Lee, Martin Man Chun Chui, Mullin Ho Chung Yu, Jasmine Lee Fong Fung, Christopher Chun Yu Mak, Christy Shuk-Kuen Chau, Ka Ka Siu, Jacqueline Hung, Kit San Yeung, Anna Ka Yee Kwong, Christopher O'Callaghan, Yu Lung Lau, Chun-Wai Davy Lee, Brian Hon-Yin Chung, So-Lun Lee

**Affiliations:** ^1^ Department of Paediatrics and Adolescent Medicine, School of Clinical Medicine, LKS Faculty of Medicine, The University of Hong Kong, Pok Fu Lam, Hong Kong SAR, China; ^2^ Department of Paediatrics and Adolescent Medicine, Queen Mary Hospital, Pok Fu Lam, Hong Kong SAR, China; ^3^ UCL Great Ormond Street Institute of Child Health, UCL and GOSH NIHR BRC, London, United Kingdom; ^4^ Department of Paediatrics and Adolescent Medicine, Duchess of Kent Children’s Hospital, Pok Fu Lam, Hong Kong SAR, China

**Keywords:** exome sequencing, genome sequencing, early-onset bronchiectasis, transmission electron microscopy, high-speed video microscopy, primary ciliary dyskinesia

## Abstract

Bronchiectasis is the abnormal dilation of the airway which may be caused by various etiologies in children. Beyond the more recognized cause of bacterial and viral infections and primary immunodeficiencies, other genetic conditions such as cystic fibrosis and primary ciliary dyskinesia (PCD) can also contribute to the disease. Currently, there is still debate on whether genome sequencing (GS) or exome sequencing reanalysis (rES) would be beneficial if the initial targeted testing results returned negative. This study aims to provide a back-to-back comparison between rES and GS to explore the best integrated approach for the functional and genetics evaluation for patients referred for assessment of bronchiectasis. In phase 1, an initial 60 patients were analyzed by exome sequencing (ES) with one additional individual recruited later as an affected sibling for ES. Functional evaluation of the nasal nitric oxide test, transmission electron microscopy, and high-speed video microscopy were also conducted when possible. In phase 2, GS was performed on 30 selected cases with trio samples available. To provide a back-to-back comparison, two teams of genome analysts were alternatively allocated to GS or rES and were blinded to each other’s analysis. The time for bioinformatics, analysis, and diagnostic utility was recorded for evaluation. ES revealed five positive diagnoses (5/60, 8.3%) in phase 1, and four additional diagnoses were made by rES and GS (4/30, 13%) during phase 2. Subsequently, one additional positive diagnosis was identified in a sibling by ES and an overall diagnostic yield of 10/61 (16.4%) was reached. Among those patients with a clinical suspicion of PCD (n = 31/61), the diagnostic yield was 26% (*n* = 8/31). While GS did not increase the diagnostic yield, we showed that a variant of uncertain significance could only be detected by GS due to improved coverage over ES and hence is a potential benefit for GS in the future. We show that genetic testing is an essential component for the diagnosis of early-onset bronchiectasis and is most effective when used in combination with functional tools such as TEM or HSVM. Our comparison of rES vs. GS suggests that rES and GS are comparable in clinical diagnosis.

## Introduction

Bronchiectasis is the abnormal dilation of the airway causing clinical symptoms such as persistent or recurrent bronchial infection, inflamed airways, and airway obstruction. It is responsible for the significant loss of lung function, significant morbidity, and early mortality if not treated correctly (Metersky et al., 2022). Bronchiectasis is a common phenotype for various diseases and has the potential to be the starting point for functional and genetic investigation for the underlying pathological diseases. Beyond bacterial and viral infections or primary immunodeficiencies, bronchiectasis could also be caused by genetic conditions such as cystic fibrosis (CF) and primary ciliary dyskinesia (PCD). CF is an inherited disorder that causes sticky mucus to build up in the respiratory airway and digestive system caused by mutations in the *CFTR* gene (Southern et al., 2007), and PCD is a rare genetic condition characterized by ciliary defects, causing ineffective ciliary movement that causes impaired mucociliary clearance. The prevalence of PCD is currently estimated to be approximately 1 in 10,000 to 1 in 15,000 in European ethnicity with an increased prevalence of approximately 1 in 2,200 births for a highly consanguineous population (Afzelius and Stenram, 2006; O'Callaghan et al., 2010). Persistent symptoms typically arise during early infancy for PCD, but it is often diagnosed in late childhood or adulthood (Kuehni et al., 2010). A major contributing factor to the missed or late diagnosis is the lack of a “one-size-fit-all” diagnostic test.

Current approaches to the clinical diagnosis of PCD involve multiple diagnostic investigations. International diagnostic guidelines published by the European Respiratory Society (ERS) in 2017 and American Thoracic Society (ATS) in 2018 recognize a combination of several examinations required for diagnosis including nasal nitric oxide concentration (nNO), transmission electron microscopy (TEM), high-speed video microscopy analysis (HSVM), and genetic testing (Lucas et al., 2017; Shapiro et al., 2018). The genetic etiology of PCD follows a monogenic rare Mendelian disease pattern typically inherited in an autosomal recessive manner. Between 1999 and 2010, PCD-causing mutations were only described in 11 genes (Knowles et al., 2013). Currently, over 50 genes have been reported as disease-causing for PCD (Wheway et al., 2021). Approximately 85% of disease-causing mutations identified are loss-of-function mutations including nonsense, frameshift insertion or deletions, canonical splice-site variants, and copy number variants (CNV). Multiple studies have identified CNV in PCD-related genes which contribute to a genetic diagnosis. Furthermore, a 27,748-bp large CNV deletion in *DRC1* and a 3,549-bp large deletion in *DNAAF4* were reported as founder mutations in East Asian and Irish populations, respectively (Zariwala et al., 1993; Keicho et al., 2020). At this time, there is no consensus by the ERS and ATS guidelines on which genes should be evaluated for PCD, and current commercial panels are not inclusive of all genes associated with PCD (Shoemark et al., 2019). Each diagnostic test is predicted to miss a proportion of cases; for example, standard electron microscopy may miss up to 30%, hence the importance of utilizing multiple diagnostic tests during investigations (Fassad et al., 2020). This is mainly due to the high genetic heterogeneity nature and the varying presentation of PCD.

Exome sequencing (ES) is more widely used than genome sequencing (GS) in the current genetic practice of Hong Kong due to the lower cost while covering the majority of pathogenic variants in the exonic regions (Tsang et al., 2019; Chung et al., 2020). Even though ES is a strong diagnostic tool, a large proportion of individuals persist as genetically undiagnosed. Negative ES results can be explained by several reasons including mosaicism, poor coverage of the exonic region, poor understanding of the genetic heterogeneity of the rare disease, lack of pathogenic evidence for detected variants, and variants that remain undetectable via ES such as CNVs, SVs, and deep intronic mutations (Frésard and Montgomery, 2018). Multiple strategies have been suggested to tackle situations where exome-negative results arise including exome sequencing reanalysis (rES), GS, long-read sequencing, transcriptome, metabolome, RNA sequencing, or investigate other cell types.

Among the several strategies suggested, rES is the most accessible and inexpensive method to use because there is no additional sequencing cost required. Studies have shown that rES is an effective measure of increasing diagnostic yield ranging from 6 to 47% (Stark et al., 2019; Fung et al., 2020). An intrinsic limitation to rES as a strategy is that rES can only analyze the variants already detected within the protein-coding region. Moreover, owing to the performance variations in the library capturing kit and sequencing instrument, there will be regions with poor uniform coverage. GS provides the capability to detect variants in regions poorly captured by ES. Approximately 30,000 rare variants can be observed in each individual with possible important impacts on gene expressions or altering conventional splicing patterns (Li et al., 2017). However, while in theory, GS is the complete sequencing strategy with the ability to detect deep non-coding variants, utilizing GS as a second-tier test for a negative ES is not as well-studied.

Since a comprehensive assessment of suspected early-onset bronchiectasis is not available in Hong Kong, we examined and evaluated the functional investigations and genetic results of 61 patients referred for assessment of bronchiectasis. Additionally, a back-to-back utility comparison between GS and rES was explored to determine the optimal strategy following an initial exome-negative result.

## Materials and Methods

### Patient Recruitment

From 2015 to 2020, a total of 61 individuals with suspected bronchiectasis evaluated according to the pediatrics respirology team in Queen Mary Hospital (QMH) were recruited into the study, which included one additional patient recruited during phase 2 as a potentially affected sibling. Participants were selected based on one of the following symptoms: 1) early-onset bronchiectasis confirmed by high-resolution computed tomography (HRCT) thorax with suggestive clinical features; 2) chronic suppurative lung disease, defined by a prolonged (>6 weeks) moist or productive cough, features of reactive airway disease, growth failure, recurrent chest infections, adventitious sound, or lung hyperinflation; and 3) “difficult to control” asthma as it has been shown that symptoms attributed to childhood asthma that are atypical or which respond poorly to conventional treatment. Specifically, school-aged children requiring step 4 or above management and step 3 or above management in pre-school age children according to the British Guideline on Management of Asthma, after excluding causes such as faulty inhaler technique, poor compliance, and environmental factors. Written informed consent was obtained from the patient’s parent or guardian to participate in the study. All eligible families were invited for next-generation sequencing. Functional investigations were offered when possible. Ethics approvals were granted by the Institutional Review Board, the University of Hong Kong (HKU)/Hospital Authority Hong Kong West Cluster (UW12-211; UW18-520).

### Functional Investigation

Functional investigation of nNO, TEM, and HSVM was performed at the Department of Paediatrics and Adolescent Medicine, at HKU and QMH as previously published (Lam et al., 2021). Sweat test and immune function workup including immunoglobulin levels were performed for all cases, and other appropriate investigations were performed if indicated. Nasal NO concentrations were measured utilizing the non-velum closure techniques in parts per billion (ppb). The nNO results were compared against the ranges previously published for healthy and PCD patients (Marthin and Nielsen, 2013). Two measurements of TEM and HSVM of the intact ciliary axoneme were targeted for this study. TEM and HSVM results were compared against the normal age-related reference range established in healthy children and adults in Hong Kong (Lee et al., 2020). Measurements and data analysis for both TEM and HSVM were performed by a trained laboratory technician.

### Next-Generation Sequencing

Exome sequencing was performed either in-house at the Department of Paediatrics and Adolescent Medicine, HKU, or performed at the Centre of PanorOmic Sciences (CPOS), HKU. In-house library preparation was prepared using either Illumina’s TruSeq™ Exome Library Prep Kit or Nextera™ Exome Kit Illumina according to manufacturer instructions. DNA libraries were then sequenced by the in-house NextSeq500 with a targeted ES depth coverage of 100×. Library preparation for ES samples sent to CPOS was prepared based on xGen Exome Research Panel v1.0 and sequenced using the Illumina NovaSeq 6000. Family-based ES was performed whenever possible. An average of 90 × depth coverage was targeted for ES sequenced at CPOS. Library preparation and sequencing for GS were performed at the Centre of PanorOmics Service, HKU. All of the libraries were prepared based on the KAPA Hyper Prep Kit (KR0961-V1.14) according to manufacturer instructions. An average of 30× coverage was targeted for GS.

### Back-to-Back Utility Comparison Between Exome Reanalysis and Genome Sequencing

After the phase 1 study, which included an initial round of 60 exome analyses, 30 exome-negative samples were recruited for genome sequencing in phase 2, to perform a back-to-back comparison investigating the diagnostic utility between rES and GS. The back-to-back comparison methodology was modified from Tan et al. (2019). NGS data were analyzed separately by two teams comprising a pair of genome analysts. Each team was blinded to each other’s analyses. The genome analysts discussed within their teams and provided a consensus result for the analysis. Consensus results were then used to calculate the diagnostic utility. Alternating rES and GS were given to each team to remove potential bias arising from different exome sequencing library kits and differing levels of variant pathogenicity assessment experience. Results including coverage comparison, number of variants detected, bioinformatics pipeline time taken, genomic analysis time taken, and overall diagnostic yield change were evaluated.

### Bioinformatics Pipeline

An in-house developed bioinformatics pipeline was used for the processing of raw ES and GS FASTQ files following the Genome Analysis ToolKit’s (GATK) best practices for germline single nucleotide polymorphisms and insertions/deletions in whole genome and exomes as previously reported (McKenna et al., 2010; Tsang et al., 2020). Raw reads were mapped to the hg19 reference human genome using the Burrows–Wheeler Aligner (BWA v.0.7.15) (Li and Durbin, 2009). Variant calling was performed using the GATK v.3.4-46 HaplotypeCaller. The same variant calling pipeline was used for the initial ES, GS, and rES cases. The analysis-ready variants for the initial ES with raw SNPs and Indels VCF file were annotated using Annotation Variation (ANNOVAR Build 20180708) (Wang et al., 2010). The variants from rES and GS were annotated using ANNOVAR Build 20200223. Variant annotation included allelic frequency from 1000 genome project, Exome Aggregation Consortium (ExAC), and Genome Aggregate Database (gnomAD v.2.1.1); in-silico prediction scores Sorting Intolerant from Tolerant (SIFT), PolyPhen2, Combined Annotation Dependent Depletion (CADD), and Rare Exome Variant Ensemble Learner (REVEL). Peddy were performed for quality control validating family inheritance and removal of duplicated samples (Pedersen and Quinlan, 2017). A virtual gene panel was used for the evaluation of genetic mutations in PCD patients from Genomics England PanelApp Primary Ciliary Dyskinesia v1.29 containing 139 genes (Martin et al., 2019).

For genome sequencing data, an additional bioinformatics pipeline was used to call CNV or SV variants. Variant calling of CNV followed the recommendations from Trost et al. (2018). The generation of CNV variants was performed using ERDS v.1.1 and CNVnator v.0.4.1 (Abyzov et al., 2011; Zhu et al., 2012). A concordant CNV VCF file was generated by merging the ERDS VCF file with the CNVnator VCF file using BEDTools v.2.27.1 set at 50% reciprocal overlap (Quinlan and Hall, 2010). Variant calling of SV was performed as previously described, if only one pathogenic/likely pathogenic variant or variant of uncertain significance (VUS) was identified (Yu et al., 2020). SV variants were called using MANTA v.1.6.0 and LUMPY v.0.2.13 (Layer et al., 2014; Chen et al., 2016). Anomalous read-pairs defined as paired ends reads that map to two different chromosomes with an abnormal insert-size or unexpected strand orientation were selected for breakpoint analysis. SpliceAI, a neural network machine learning–based algorithm to predict splicing variants, was performed on samples when one pathogenic/likely pathogenic variant or VUS was identified in a gene (Jaganathan et al., 2019). CNV and SV variant annotation was performed using Annotation and Ranking of Human Structural Variations (AnnotSV v.2.2). Manual inspection of the CNV and SV identified by the bioinformatics pipeline was performed on Integrative Genomics Viewer (IGV).

**FIGURE 1:**
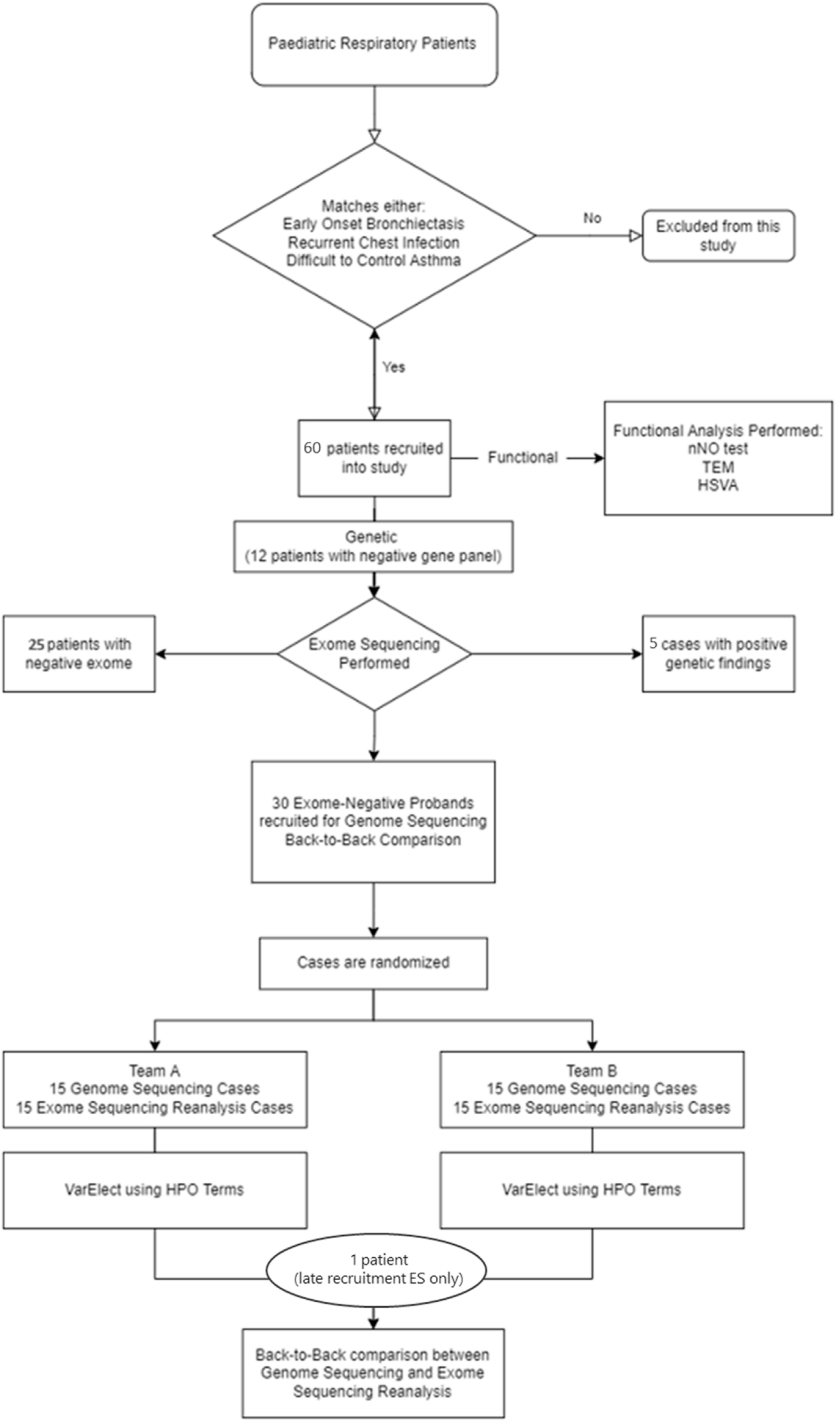
Diagnostic workflow. The diagram shows this study design and workflow. In phase 1, 61 patients were recruited into the study for genetic and functional analysis. In phase 2, 30 exome-negative probands were recruited for GS and rES back-to-back comparison analysis. HSVM = high-speed video microscopy; HPO = human phenotype ontology; nNO test = nasal NO test; TEM = transmission electron microscopy.

The pathogenicity interpretation of rare SNP and Indel genetic variants was performed following the American College of Medical Genetics (ACMG) Variant Interpretation guidelines (Richards et al., 2015). In addition, loss-of-function variants were classified utilizing the modified recommendations from the ClinGen Sequence Variant Interpretation Working Group (Abou Tayoun et al., 2018). The ACMG guideline modifications utilizing a Bayesian framework were also used in this study to evaluate the likelihood of pathogenicity in VUS cases (Tavtigian et al., 2018). Pathogenicity interpretation of CNVs was classified according to the ACMG/ClinGen guidelines (Riggs et al., 2020). The full study workflow can be seen in Figure 1.

## Results

**Cohort Characteristics:** A total of 61 participants were recruited into the study for exome sequencing, which constituted 33 males and 28 females. Twelve participants previously had gene panel genetic testing (*CCDC103*, *CCDC114*, *CCDC39*, *CCDC40*, *CFTR*, *DNAAF1*, *DNAAF2*, *DNAAF3*, *DNAH11*, *DNAH5*, *DNAI1*, *DNAI2*, *DNAL1*, *HEATR2*, *INVS*, *LRRC6*, *NME8*, *OFD1*, *RPGR*, *RSPH4A*, and *RSPH9*) with negative results. Age at recruitment ranged from 1 to 32 years old (median: 11 years old). Fifty-two patients (85.2%) were below 18 years at the recruitment. Cases were recruited as trios (50.8%) if available. The remaining families were sequenced as singleton (13.1%), duos (27.9%), and quadruple (8.2%). Three cases were recruited into the study with a possible affected sibling due to the similar clinical features and positive familial genetic results. Two families recruited in this study were consanguineous (Case 5 and 12). All samples passed sample-level quality control, that is, with correct familial inheritance pattern and sexes within each family and no duplicated samples were found in the cohort. Thirty cases from phase 1 were recruited for GS in phase 2. Participants with trio-based sequencing data available and relevant functional results including low nNO, TEM defects, low ciliary beat frequency, or abnormal ciliary beat pattern by HSVM, were prioritized for GS. The majority of patients (*n* = 28; 93.3%) recruited were below 18 years at the initial age of recruitment with 13 males and 17 females. Two duos (6.7%) cases were recruited into GS due a clinical history strongly suggestive of PCD. Supplementary Table S1 lists all the PCD participant characteristics for both initial ES and GS. Supplementary Table S2 lists the clinical features and molecular diagnosis for all 61 patients. Supplementary Table S3 lists the number of SNP and indels variants generated by rES and GS annotated and grouped by ANNOVAR.

### Phase 1 and Phase 2 Analyses

#### Phase 1 Exome Sequencing

Sixty participants underwent ES as the first-tier diagnostic test in phase 1 of this study. ES analysis was performed from 2019 to 2020. A total of five individuals received a positive genetic diagnosis which corresponds to an exome sequencing diagnostic yield of 5/60 (8.3%), and none of them have been diagnosed by other tests.

#### Phase 2 Back-to-Back rES and GS Analysis

Phase 2 which included the back-to-back rES and GS analysis was performed from January to June 2021. Four individuals received a positive genetic diagnosis during phase 2 of the study (*DNAI1*, *DNAH11*, and *DNAH5* x2). These four positive genetic diagnoses could only be made during phase 2 by both rES and GS due to updated literature. While the back-to-back analysis comparison was taking place, case 69 (sibling of the case 59) was recruited into the ES study. An additional positive diagnosis was made for case 69 (*DNAH11*) by ES, which at the same time led to an additional diagnosis for case 59.

#### Diagnostic Yield

Diagnostic yield for phase 1 was 8.3% (*n* = 5/60) and diagnostic yield for phase 2 was 13.3% (*n* = 4/30). Combining all diagnoses, a diagnostic yield of 10/61 (16.4%) was obtained. The ten participants with a positive genetic diagnosis had a total of 12 unique mutations in seven genes that met the pathogenic or likely pathogenic criteria according to the ACMG classification. No clinically relevant CNVs/SVs were detected in this study. Table 1 lists the full details with patient’s clinical history, genetic mutation, pathogenic classification, and functional results.

**TABLE 1:** Comprehensive functional and genetic landscape of early-onset bronchiectasis patients in Hong Kong.

Case	Clinical history	Gene	Allele 1 (ACMG classification)	Allele 2 (ACMG classification)	Phase	Family type	nNO (ppb)	TEM	HSVM
6	Bronchiectasis, situs inversus, dextrocardia, sinusitis, and bilateral otitis media	*CCDC40*	NM_017950.4(*CCDC40*)	Homozygous	1	Duos (Father)	12	Disarranged cilia	Slow-moving and dyskinetic
			c.626_627del						
			p. (Gly209GlufsTer59)						
			[likely pathogenic]						
12	History of *pseudomonas aeruginosa* infection, recurrent bronchiolitis, bilateral bronchiectasis	*CFTR*	NM_000492.4(*CFTR*)	Homozygous	1	Singleton^a^	91	Normal	Normal
			c.274-1G>C						
			[pathogenic]						
36	Bronchiectasis, allergic rhinitis, history of pneumonia, sinopulmonary infection	*RSPH4A*	NM_001010892.3 (*RSPH4A*)	Homozygous	1	Trios	19	Central microtubules defect	Circular beat pattern
			c.1774_1775delTT						
			p. (Leu592AspfsTer5)						
			[likely pathogenic]						
61	Bronchiectasis, history of *Pseudomonas aeruginosa* infection, history of *Staphylococcus aureus* infection, mixed restrictive and obstructive disease	*CFTR*	NM_000492.4(*CFTR*)	NM_000492.4(*CFTR*)	1	Duos (Father)	N/A	N/A	N/A
			c.2912_2948del	c.1766+5G>T					
			p. (Ile972MetfsTer16)	Unknown					
			Paternal	[pathogenic]					
			[pathogenic]						
63	Dextrocardia, situs inversus, congenital pneumonia, persistent nasal discharge	*DNAH11*	NM_001277115.2 (*DNAH11*)	Homozygous	1	Trios	N/A	Normal	Static beat pattern
			c.11749_11752delGTTA						
			p. (Val3917LysfsTer20)						
			[likely pathogenic]						
2	Chronic suppurative lung disease, bronchiectasis, rhinitis, recurrent chronic bilateral otitis media	*DNAI1*	NM_012144.4 (*DNAI1*)	NM_012144.4 (*DNAI1*)	2	Trios	25	Outer dynein arm defect	Slow-moving and dyskinetic
			c.634C>T	c.1355_1357del					
			p. (Gln212Ter)	p. (Phe452del)					
			Maternal	Paternal					
			[likely pathogenic]	[likely pathogenic]					
5	Biliary atresia, dextrocardia, situs inversus with normal heart structure, chronic lung disease	*DNAAF3*	NM_178837.4 (*DNAAF3*)	Homozygous	2	Trios ^a^	N/A	Outer dynein arm and inner dynein arm defect	Immotile cilia
			c.493G>C						
			p. (Gly165Arg)						
			[VUS]						
25	Bilateral bronchiectasis, severe necrotizing pneumonia, mixed obstructive and restrictive lung disease with insignificant bronchodilator response, pneumothorax, recurrent chest infection, previous tracheostomy performed	*DNAH9*	NM_001372.4 (*DNAH9*)	NM_001372.4 (*DNAH9*)	2	Trios	N/A	Normal	Slow-moving
			c.3648delG	c.5093G>A					
			p. Ala1217GlnfsTer4	p. (Gly1698Asp)					
			Paternal [likely pathogenic]	Maternal					
				[VUS]					
54	Bronchiectasis-suspected small airway disease, bronchiolitis, complicated with cystic bronchiectasis, right upper lobes, and right lower lobes medial segment collapse, pneumothorax, recurrent wet cough	*CCNO*	NM_021147.5(*CCNO*)	Homozygous	2	Trios	N/A	Only non-ciliated epithelia seen	Few cilia seen
			c.788G>C						
			p.Arg263Pro						
			[VUS]						
59	Recurrent sinopulmonary infections, recurrent right middle lobes consolidation and collapse, persistent *Haemophilus influenzae* infection, bronchiectasis	*DNAH11*	NM_001277115.2 (*DNAH11*)	NM_001277115.2 (*DNAH11*)	2	Quadruple	N/A	Normal	Static
			c.3426-1G>A	c.10264G>A					
			Maternal	p. (Gly3422Arg)					
			[pathogenic]	Paternal					
				[likely pathogenic]					
62	Bronchiectasis in bilateral lower lobes, complete collapse of right middle lobe, right bronchomalacia with *Haemophilus influenzae*, right middle lobe pneumonia	*DNAH5*	NM_001369.3 (*DNAH5*)	NM_001369.3 (*DNAH5*)	2	Quadruple	N/A	Inconsistent results	N/A
			c.10438G>T	c.4355+5G>A					
			p. (Glu3480Ter)	Paternal					
			Maternal	[likely pathogenic]					
			[pathogenic]						
64	Daily wet cough, perinatal pneumonia, and right middle lobe pneumonia, active bilateral airway inflammation, severe bronchomalacia with complete collapse right middle lobe, lower left lobe bronchi, bronchiectasis with sputum retention, tonsil and adenoid hypertrophy, bronchial wall thickening of central airways of bilateral lower lobes	*DNAH5*	NM_001369.3 (*DNAH5*)	NM_001369.3 (*DNAH5*)	2	Quadruple	N/A	Outer Dynein Arm defect	N/A
			c.10438G>T	c.4355+5G>A					
			p. (Glu3480Ter)	Paternal					
			Maternal	[likely pathogenic]					
			[pathogenic]						
69	Sinusitis, mile bronchiectasis, suspected diffuse pan bronchiolitis, suggestive of mucus impaction and endobronchial spread of infection	*DNAH11*	NM_001277115.2 (*DNAH11*)	NM_001277115.2 (*DNAH11*)	2	Quadruple	N/A	N/A	N/A
			c.3426-1G>A	c.10264G>A					
			Maternal	p. (Gly3422Arg)					
			[pathogenic]	Paternal					
				[likely pathogenic]					

a Consanguineous parent.

In addition, we retrospectively classified 31/61 patients in our cohort of suspected bronchiectasis into those with clinically suspicious of PCD, based on the ERS guidelines (Lucas et al.). Limited by the lack of nNO for majority of patients, only clinical features were being investigated. Patients with two or more of the recommended six clinical features from the ERS guidelines were classified as those clinically suspicious of PCD. The six clinical features included 1) persistent wet cough, 2) situs anomalies, 3) congenital cardiac defects, 4) persistent rhinitis, 5) chronic middle ear disease with or without hearing loss, and 6) a history in term infants of neonatal upper and lower respiratory symptoms or neonatal intensive care admittance. We also included any patients with a confirmed PCD diagnosis in a sibling prior to the study. Among the 31 patients with clinical suspicion of PCD, the diagnostic yield was 26% (*n* = 8/31). In contrast, for patients who did not fulfill the clinical criteria of PCD, no PCD-related molecular diagnosis was obtained, and CF was diagnosed in two patients by the presence of biallelic CFTR mutations.

### Integrated Approach in a Cohort of Suspected Bronchiectasis

In addition to the main findings, we report cases that illustrate the need for an integrated approach to a cohort of suspected bronchiectasis, which is demonstrated by 1) a case of CF bronchiectasis, 2) a case of normal ciliary ultrastructure that would have been missed if genetic testing was not done, as well as 3) two cases where VUS may highlight the genotype-phenotype correlation between the genetic and functional results, and 4) one case to illustrate the advantage of increased coverage in GS.

#### CF Bronchiectasis

**FIGURE 2:**
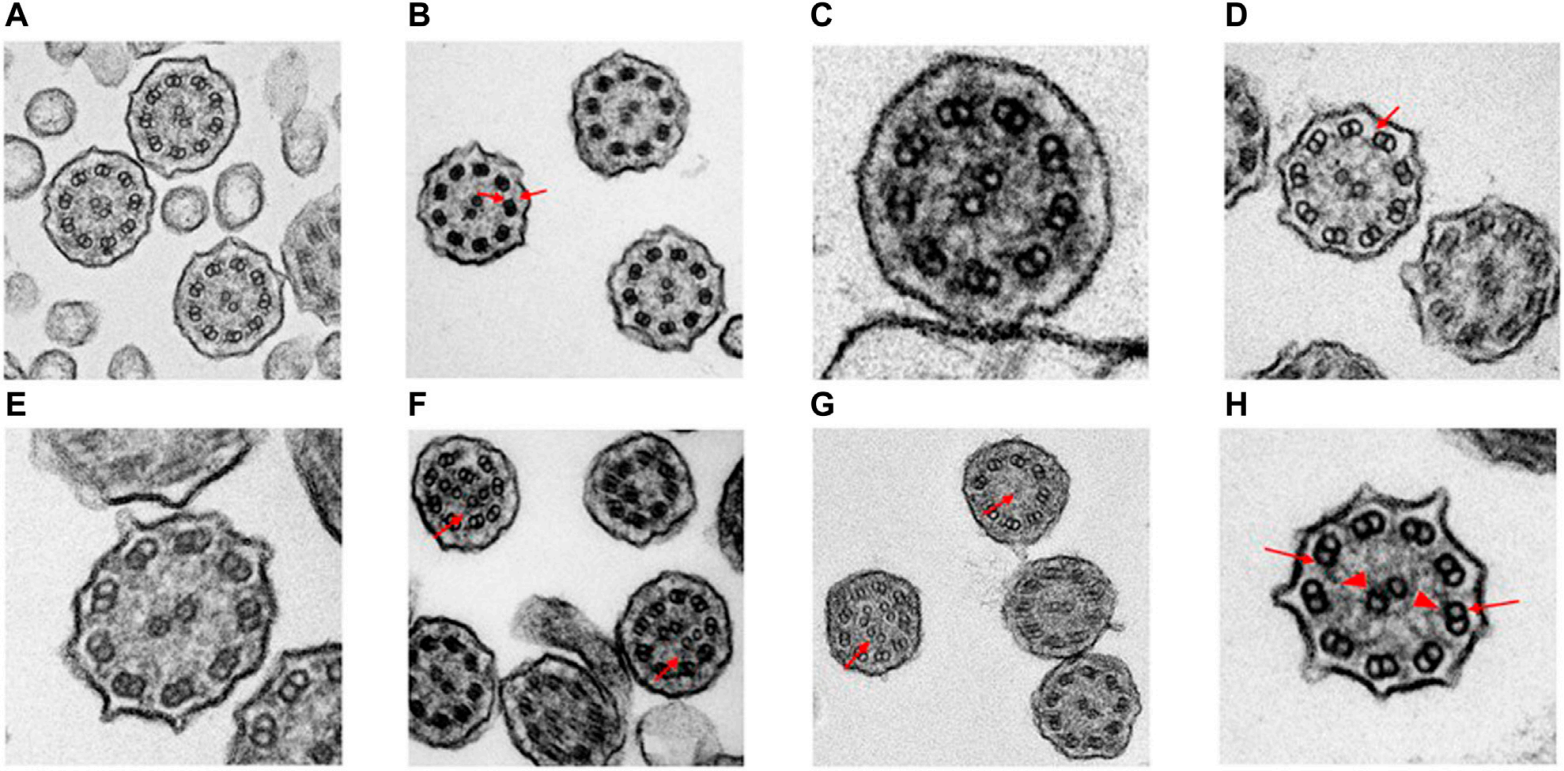
Transmission electron microscopy results of suspected early-onset bronchiectasis patients in Hong Kong. **(A)** Case 12—Confirmed CF case showed a normal ciliary ultrastructure. **(B)** Case 5—A homozygous recessive *DNAAF3* c 493G>C variant was identified. TEM results showed an outer and inner dynein arm (arrows) defect which is similar to previously reported cases **(C)**. Case 25 cilia proximal arm—A compound heterozygous variants could be seen in *DNAH9.* The literature has shown that subtle outer dynein arm defect can be seen in TEM. The ciliary ultrastructure analysis in the proximal arm shows a normal ultrastructure **(D)**. Case 25 cilia distal arm shows a subtle outer dynein arm (arrow) defect. (E) Case 63—TEM results showed a normal ciliary ultrastructure. A pathogenic homozygous recessive *DNAH11* c.11749_11752delGTTA was identified and confirmed the clinical diagnosis despite the normal ultrastructure. HSVM showed a static beat pattern in 50% of cilia samples **(F)**. Case 6—TEM results showed that 47% of cilia were in a disarranged pattern (arrows). This matches the literature of loss-of-function mutations in the *CCDC40* gene. The frameshift deletion detected was *CCDC40* c.626_627del. **(G)**. Case 36—A trio-based ES revealed a frameshift deletion mutation on the *RSPH4A* gene, c.1774_1775del. Two separate TEM results showed 43 and 35% of ciliary samples with missing/extra-central microtubules (arrows). Previously reported literature has shown that patients with *RSPH4A* mutations are also presented with circular beating patterns and cilia without central microtubules **(H)**. Case 2—A compound heterozygous of two variants detected in trans within the *DNAI1* gene was seen in both trio-based rES and GS. ODA (arrows) defect was also detected in the TEM investigation which showed an average of 3.4 outer dynein arms detected per cilia (arrowheads, inner dynein arm). (bar = 100 nm).

Case 12: A 4-year-old South Asian male from a consanguineous family presented with clinical symptoms of bronchiolitis, bilateral bronchiectasis, and history of *Pseudomonas aeruginosa* infection. Singleton-ES showed a homozygous splice-site mutation, NM_000492.4(*CFTR*):c.274-1G>C. Subsequent Sanger sequencing showed both parents were heterozygous for the variant. The *CFTR* gene is a well-known gene with a strong genotype–phenotype correlation with cystic fibrosis (OMIM #219700). This splice mutation was previously reported as pathogenic on ClinVar (accession: VCV000048680.12) and the following two positive sweat tests of 91.2 and 107.1 mmol/L confirmed the cystic fibrosis diagnosis. Functional examination confirms our understanding of the diagnosis as cystic fibrosis patients are not presented with any changes in ciliary ultrastructure or beat pattern and frequency. Figure 2A shows normal ciliary ultrastructure from the TEM examination. The reduced nNO test of 91 ppb is concurrent with the current understanding of nitric oxide levels in cystic fibrosis patients (Michl et al., 2013).

#### PCD with Normal Ciliary Ultrastructure

Case 59: A 17-year-old East Asian female presented with recurrent sinopulmonary infections, recurrent right middle lobes consolidation and collapse, persistent *Haemophilus influenzae* infection, and bronchiectasis. This case was recruited into phase 2 because phase 1 was inconclusive. A compound heterozygous of maternally inherited pathogenic canonical splice-site variant NM_001277115.2 (*DNAH11*):c.3426-1G>A and paternally inherited likely pathogenic missense variant NM_001277115.2 (*DNAH11*):c.10264G>A: p. (Gly3422Arg) was detected using both rES and GS. *DNAH11* is also a heavy-chain protein that encodes the outer dynein arm (ODA) in the anemone and is typically associated with PCD with an autosomal recessive inheritance pattern. Case 63 also had compound heterozygous pathogenic mutations in *DNAH11*. The nNO test was not performed due to COVID-19 restrictions, and TEM investigations showed a normal ciliary ultrastructure. Functional investigations for HSVM results returned confirming a functional diagnosis of PCD. The HSVM investigations further supports the diagnosis of PCD and showed a static ciliary beat pattern in 50% of cilia samples. For the remaining cilia, 46% showed normal beating and 4% showed stiff beating. Overall beat frequency is at 10.77 Hz.

To further strengthen the pathogenicity of the *DNAH11* variants, case 69, a 12-year-old younger sister of case 59, who presented with clinical symptoms of sinusitis, mild bronchiectasis, suspected diffuse pan bronchiolitis, suggestive of mucus impaction, and endobronchial spread of infection, was recruited during phase 2 of our study. Although no functional assessments were performed due to the emergence of COVID-19, genetic results showed that the same segregation of c.3426-1G>A and p.(Gly3422Arg) variants were detected in case 69. This further increases the pathogenicity classification of the mutations due to the familial segregation of the disease-causing variants.

#### Functionally Relevant PCD Cases in Cases with Variant of Unknown Significance

Case 25: A 21-year-old East Asian female with clinical symptoms of bilateral bronchiectasis, severe necrotizing pneumonia, mixed obstructive and restrictive lung disease with insignificant bronchodilator response, pneumothorax, and recurrent chest infection was recruited into the study. Initial ES detected a compound heterozygous mutation in the *DNAH9* gene including a paternally inherited frameshift NM_001372.4 (*DNAH9*):c.3649del: p. (Ala1217GlnfsTer4) and a maternally inherited missense NM_001372.4 (*DNAH9*):c.5093G>A: p. (Gly1698Asp) variant. Both rES and GS were able to detect the two variants. *DNAH9* is a gene recently discovered in 2018 with a genotype–phenotype correlation with PCD, and is inherited in an autosomal recessive manner. The frameshift p.(Ala1217GlnfsTer4) and missense p.(Gly1698Asp) variants were classified as likely pathogenic and VUS, respectively, according to the ACMG guidelines. There were no changes in ACMG classification since the initial ES analysis.

Functional investigations were performed for this case and showed a strong genotype–phenotype correlation. TEM results showed an average of 7.7 outer and 7.8 inner dynein arm count which suggests a normal ciliary ultrastructure (Figure 2C). However, TEM photos of the distal axoneme showed ciliary ultrastructure lacking in the outer dynein arm (Figure 2D). The HSVM investigations showed slow-moving cilia beat frequency at 6.5 Hz. A total of 98% of the cilia showed normal beating, while 2% were immotile cilia. There was no obvious stiffness at the base of the cilia. Despite the functional investigations matching the genetic findings, the variant was not upgraded to likely pathogenic due to the stringent nature of ACMG criteria.

Case 54: An 18-year-old East Asian female presented with bronchiectasis, suspected small airway disease, bronchiolitis, complicated with cystic bronchiectasis, right upper lobes and right lower lobes medial segment collapse, pneumothorax, and recurrent wet cough was recruited into the study. Initial ES results were negative, and this case was subsequently recruited into the back-to-back comparison. A homozygous missense mutation was detected in both rES and GS, NM_021147.5(*CCNO*):c.788G>C: p.(Arg263Pro). This variant was inherited from both parents. Cyclin O, also known as *CCNO*, plays an important role in the function of deuterostome-dependent amplification of basal bodies and their docking mechanism (Wallmeier et al., 2014). *CCNO* is known to have a genotype–phenotype correlation with PCD and is inherited in an autosomal recessive manner. The missense variant is considered rare with an allelic frequency of 0.00001423 in the gnomAD v2.1.1 database but was not predicted pathogenic by most bioinformatics prediction tools. Based on the current evidence, the p.(Arg263Pro) variant was classified as a VUS.

This variant was reported due to the strong genotype–phenotype correlation with the TEM and HSVM functional results. Both functional investigations showed there were a limited number of epithelial cilia that can be observed in the patient’s live ciliary sample. Current understanding of the reduced generation of motile cilia can only be seen in two genes, *MCIDAS* and *CCNO* (Boon et al., 2014). Further investigation into *MCIDAS* yielded no relevant mutations in both rES and GS. Despite the genetic result matching with the functional findings, the mutation lacked further evidence to be upgraded according to the ACMG guidelines. Further experimental methodologies would be required to upgrade the pathogenicity of this variant.

#### Benefit of GS over ES Demonstrated by Differences in Coverage of a VUS Variant

Case 5: A 5-year-old South Asian male from a consanguineous family who was recruited into the study presented with clinical symptoms of biliary atresia, dextrocardia, situs inversus with normal heart structure, and chronic lung disease. Initial ES did not detect any genetic findings in any clinically relevant genes. GS was pursued for this case, and a homozygous missense variant NM_178837.4 (*DNAAF3*):c.493G>C p. (Gly165Arg) was discovered. This variant was not detected during the initial exome sequencing due to the low coverage of the region. Mutations in *DNAAF3* cause defects in the cytoplasmic preassembly of the dynein arms and are associated with the phenotype primary ciliary dyskinesia with an autosomal recessive inheritance pattern. The variant was classified as VUS according to the ACMG guidelines as there is limited information about the effects of this mutation.

Functional investigations were performed for this case and showed a strong genotype–phenotype correlation. The TEM results for case 5 showed an ODA + inner dynein arm (IDA) defect (Figure 2B). The HSVM investigations showed completely immotile cilia beat frequency that was confirmed on retesting. Literature has shown that PCD patients with *DNAAF3* mutations present with similar phenotypes (Mitchison et al., 2012). Despite the functional investigations matching the genetic findings, the variant was not upgraded to likely pathogenic due to the stringent ACMG criteria and the scarcity of medical literature on the gene and the functional investigations.

## Discussion

In this study, we performed ES on 61 patients referred for assessment of bronchiectasis, which included patients with suspected early-onset bronchiectasis, recurrent chest infections, and difficult-to-control asthma in Hong Kong. Cases with trio samples available, strong clinical phenotypes, or functionally relevant investigations were recruited for GS. A back-to-back comparison between rES and GS was performed on 30 initially negative exome cases. There is an increase in diagnostic yield with rES but with minimal additional increase over rES with GS.

The virtual gene panel used for the evaluation of genetic mutations in PCD patients was obtained from Genomics England PanelApp Primary Ciliary Dyskinesia v1.29 containing 139 genes. Within the 139–virtual-gene panel screened, 135 genes screened had at least 10 × read depth across the exonic region in over 90% of the PCD rES results (Supplementary Figure S1). Notable exceptions included *BBS5*, *CLRN1*, *CRX*, and *GDF1*. With the exception of the *RPGR* gene, 138 genes met 10 × read depth across the exonic region in over 90% of the GS samples requirement. *RPGR* is notably a gene that is listed as a diagnostic-grade green (strong confidence) genotype–phenotype correlation with PCD.

An overall diagnostic yield of 10/61 (16.4%) was made by ES or rES and GS combined. The additional yield of rES and GS beyond the initial ES analysis was 4/30 (13.3%). The diagnostic yield of this study is not comparable to other PCD cohorts due to the different nature of the recruitment process. In our study, patients with either bronchiectasis, chronic suppurative lung disease, or “difficult to control” asthma were recruited into the study. Functional investigations were performed parallel to genetic investigations. This differs from other studies highlighted by ERS and ATS, which performs genetic investigations only on patients with strong clinical phenotypes of PCD or functionally confirmed to be PCD (Lucas et al., 2017; Shapiro et al., 2018). By performing functional investigations prior to the genetic testing, this would exclude samples with weak phenotypes. Another reason for the lower yield in ES is that 12 cases were negative from a previous gene panel analysis which included 20 PCD genes and *CFTR*. Moreover, the genetic basis of PCD is still unknown in 20–30% of confirmed cases. As a result, a lower diagnostic yield compared to other reported PCD studies is expected.

In this study, a back-to-back clinical utility comparison between rES and GS was performed. Other analysis points were also analyzed including bioinformatics pipeline time taken, HPO terms used for analysis, and genomic analysis time taken (Supplementary Tables S4, S5, S6). Both rES and GS were able to find the same genetic variants identified in phase 2; however, only GS was able to detect the VUS *DNAAF3* c.493G>C variant due to non-uniform coverage of the exons in ES. Changes in pathogenicity classification in rES cases were mainly due to updated literature, correlation with functional investigations, and improvements in in-silico bioinformatics predictions for splicing variants. Despite the added benefits of detecting deep intronic variants, intergenic variants, and CNV by GS, no functionally relevant CNV mutations were detected in this study. As CNV mutations were detected in PCD patients from other studies, the lack of detection could be attributed to the small sample size or differences in genetic etiology (Morimoto et al., 2019; Keicho et al., 2020; Wheway et al., 2021). This reduced the added benefits of GS in comparison to rES. This also demonstrated a limited benefit in proceeding to GS instead of performing rES.

**FIGURE 3:**
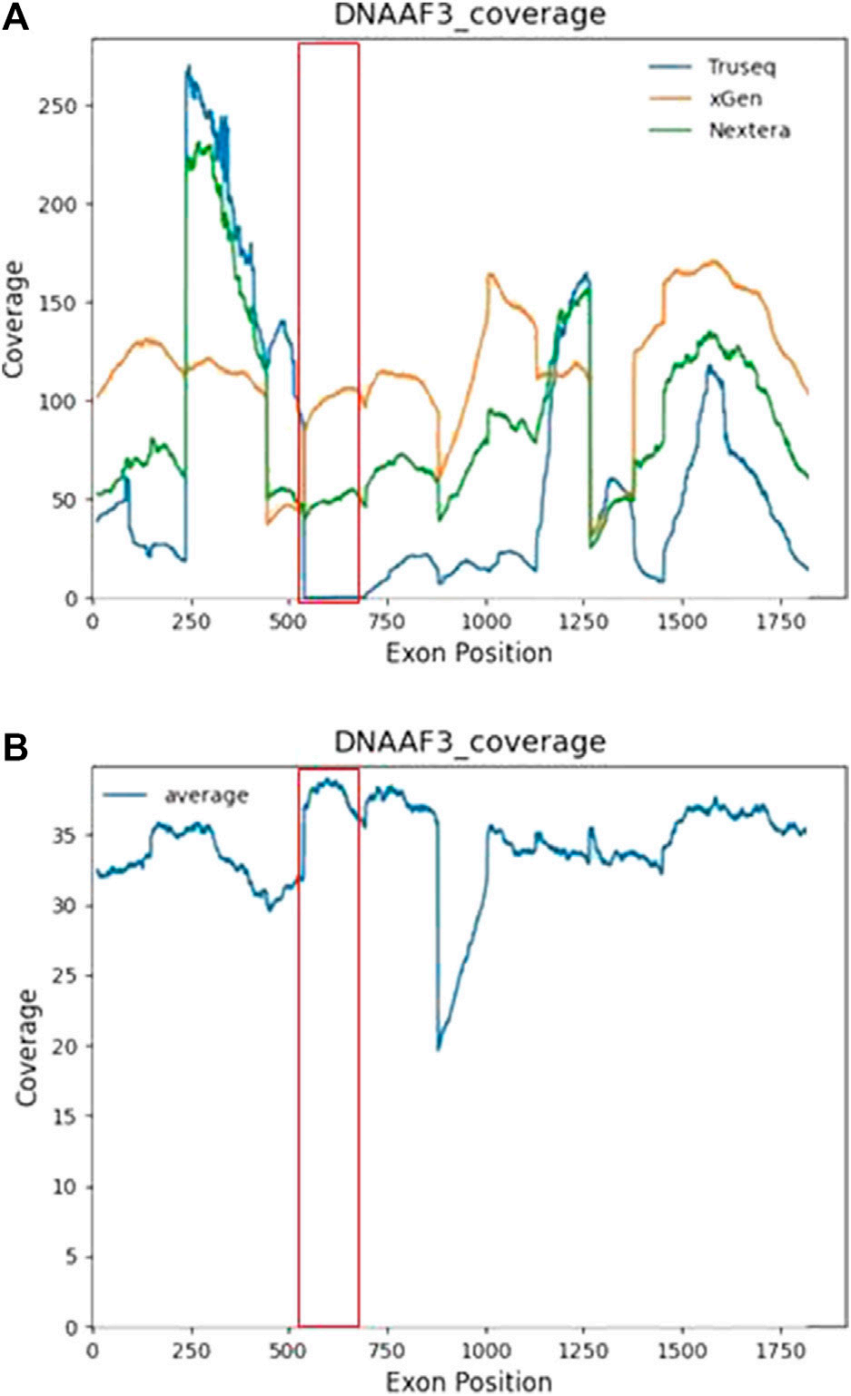
Nucleotide by nucleotide coverage of *DNAAF3* in ES and GS. The line plot shows the average coverage of each nucleotide in the *DNAAF3* gene. **(A)** Coverage of ES split across library preparation kits. Across 30 ES samples that used Truseq, an average of 0× read depth was seen for exon 5 of *DNAAF3*. **(B)** Coverage of GS across the *DNAAF3* gene.

Further examination into the ES coverage data showed uneven coverage across exons in different ES library preparation kits which explained the detection of *DNAAF3* c.493G>C exclusively in GS (Figure 3). Case 5 used the TruSeq Exome Library Prep Kit, which showed poor coverage over exon 5 in the *DNAAF3* gene. Across 30 exome samples that used TruSeq, a 0× average read depth was seen across exon 5 of *DNAAF3*. The low coverage of exon 5 of *DNAAF3* is not limited to TruSeq. In gnomAD exome coverage with 141,456 samples, the average coverage over this nucleotide is 1.7×. Newer exome library preparation kits such as xGen and Nextera have fixed the issue of low coverage in this exon and have sufficient coverage of exon 5. This case shows that despite the great utility of rES, resequencing samples should be considered if the initial sequencing was performed a few years ago, preferably with a new exome capture kit. Improvements in ES library preparation can reduce concerns such as uneven coverage.

One of the major advantages of utilizing genetic testing as a diagnostic tool in suspected early-onset bronchiectasis is the lack of a “one-size-fit-all” diagnostic test. Genetic testing was able to identify pathogenic mutations in the *DNAH11* gene in cases 59, 63, and 69. As previously mentioned, pathogenic mutations within the *DNAH11* gene do not cause structural defects identifiable from TEM investigations. For case 59, a compound heterozygous variant including a maternally inherited splicing variant c.3426-1G>A and paternally inherited missense variant p.(Gly3422Arg) were detected. The variants were both previously detected during the initial round of exome sequencing, but a genetic diagnosis was not completed due to insufficient evidence as the p.(Gly3422Arg) missense variant was classified as a VUS. The previous combination of ACMG criteria used included absent from controls (PM2), detected in trans with a pathogenic variant (PM3), and multiple lines of computational evidence support a deleterious effect on the gene or gene product (PP3). In phase 2, functional TEM and HSVM results confirmed a functional diagnosis of PCD. Similar to case 63, the TEM results returned a normal ciliary ultrastructure consistent with *DNAH11* findings and HSVM results showed a static ciliary beat pattern. The added combination of TEM and HSVM results allowed the usage of PP4 criteria, the patient’s phenotype or family history is highly specific for a disease with a single genetic etiology. The new combination of PM2, PM3, PP3, and PP4 satisfies the ACMG combination criteria of a likely pathogenic variant, thereby reaching a genetic diagnosis. The usage of combining functional and genetic results could also be seen in other cases too. Both TEM and HSVM were also useful tools to confirm the genetic diagnosis in case 63, 6, 36, and 2 (Figures 2E–H). This shows that the combination of functional examination and genetic testing is useful in the diagnosis of early-onset bronchiectasis due to the complex functional presentation of PCD.

With the rate of growth in discovering new genes for a genetically heterogeneous condition such as PCD, reanalysis is expected to be useful in helping patients reach a genetic diagnosis for their condition. Initial ES detected a compound heterozygous mutation in the *DNAH9* gene including a paternally inherited loss-of-function p.(Ala1217GlnfsTer4) and a maternally inherited missense p.(Gly1698Asp) variant. Both rES and GS were able to detect the two variants. *DNAH9* is a gene recently discovered in 2018 with an autosomal recessive inheritance pattern (Fassad et al., 2018; Shoemark et al., 2018). Due to the recent discovery of *DNAH9*, there is currently limited evidence for functional investigations and genotype–phenotype correlation. The gene is currently listed in the PanelApp gene panel under the category of “No List,” suggesting curation for this gene is yet to be completed. Patients with *DNAH9* mutations have been reported to have relatively mild or no respiratory phenotypes which contradicts the severe clinical phenotype identified in case 25 (Loges et al., 2018; Lucas et al., 2020). Furthermore, TEM results showed no ciliary ultrastructure defect which is possibly compatible with the subtle ODA defect observed in *DNAH9* cases. The lack of cases and understanding of missense variants within the *DNAH9* gene greatly restricts the pathogenicity classification. Without an abundance of cases or experimental evidence of the p.(Gly1698Asp) variant, it remains a VUS variant under the ACMG recommendations. This highlights the importance of performing genetic analysis in combination with functional tests for better genotype–phenotype correlation. In order for genes to be rated green on PanelApp, pathogenic variants need to be identified in ≥3 independent families. This emphasizes the importance of identifying and investigating early-onset bronchiectasis to increase evidence level for amber/red/no list genes. Further investigations will need to be performed and ensure the genetic diagnosis.

### Limitations

The study design focuses on suspected early-onset bronchiectasis cases which has potential non-genetic causes. While this study has effectively shown the mutational spectrum and genetic landscape of early-onset bronchiectasis patients in Hong Kong, more patients will need to be recruited to effectively show the genetic diagnosis. Genetic analysis will also need to be frequently reanalyzed due to the growing list of genes associated with PCD.

The back-to-back comparison of rES and GS in this study was limited by resources. Ideally, all exome-negative samples would have also undergone GS for a larger cohort and thus better comparison. The remaining 25 cases with negative ES result would be subject to GS when resources become available to see if there is any confounding effect. Additionally, the patient’s phenotype was not evaluated by a standardized HPO terms approach which would have utilized clinical geneticists and clinicians in standardizing the patient’s phenotype and cohort phenotype characteristics. Furthermore, a better comparison between rES and GS would ideally include patients with a wider spectrum of disease conditions. Different inheritance patterns and disease mechanism could potentially show the added benefits of detecting intronic variants, repeat expansion, CNV, and SV variants in GS over rES.

Lastly, this study was also affected by the COVID-19 pandemic. As suspected, bronchiectasis and PCD is a respiratory disease, so further patient recruitment and functional investigations were halted for safety concerns. Some samples did not undergo typical functional investigations despite reaching a genetic diagnosis. This limited the ability to correlate genetic findings with functional results.

## Conclusion

In this cohort of early-onset bronchiectasis, recurrent chest infections, and difficult-to-control asthma patients suspected of primary ciliary dyskinesia, an overall diagnostic yield of 16.4% was achieved. This study has shown that utilizing genetic testing in combination with functional investigations has increased diagnostic yield for patients with pathogenic mutations in PCD genes due to the lack of a “one-size-fit-all” diagnostic test. A back-to-back comparison between rES and GS has shown that rES and GS are comparable in clinical diagnosis. It may appear there was a little added benefit that could be seen from proceeding to GS in this study; this is due to the lack of effective means to evaluate intronic mutations and the lack of clinically relevant CNV detected in this study. While resequencing samples is recommended if the initial sequencing was performed more than a few years ago, the issue of uneven gene coverage in ES may not be completely resolved by either resequencing or reanalysis of ES. This study has shown that further investigation is required to evaluate other complementary approaches after an exome-negative result.

## Data Availability

The datasets for this article are not publicly available due to concerns regarding minor participant/patient anonymity. Requests to access the datasets should be directed to the corresponding authors. Data available from the corresponding authors will be de-identified before the data is handed over to qualified researchers.
