# Supplementary material for: Functional Evaluation and Genetic Landscape of Children and Young Adults Referred for Assessment of Bronchiectasis
Source: Front Genet. 2022 Aug 8;13:933381. doi: 10.3389/fgene.2022.933381 (PMC9393783; doi:10.3389/fgene.2022.933381)
Supplement: Supplementary file 1 [file datasheet1.pdf]

Table S1: Subject Demographic of suspected early onset bronchiectasis patients in exome sequencing and genome sequencing

	**Exome Sequencing (n=61)**		**Genome Sequencing (n=30)**	
	**Number**	**Percentage (%)**	**Number**	**Percentage (%)**
**Age**				
**<10**	28	45.9	18	60.0
**10-18**	24	39.3	10	33.3
**>18**	9	14.8	2	6.7
**Sex**				
**Male**	33	54.1	13	43.3
**Female**	28	45.9	17	56.7
**Type of family sequencing**				
**Singleton**	8	13.1	0	0.0
**Duos**	17	27.9	2	6.7
**Trios**	31	50.8	25	83.3
**Quadruple**	5	8.2	3	10.0
**Consanguinity**	2	3.3	1	3.3

Table S2: Clinical features and molecular diagnosis for all 61 patients.

Case	Recruitment Group			Clinical Features based on the ESR guidelines						Sibling with confirmed PCD prior to study	Clinically suspected PCD (2 or more features)	Molecular Diagnosis
	1	2	3	Persistent wet cough	Situs anomalies	Congenital cardiac defects	Persistent rhinitis	Chronic middle ear disease with or without hearing loss	A history in term infants of neonatal upper and lower respiratory symptoms or neonatal intensive care admittance			
**1**				✓				✓			Yes	
**2**								✓	✓		Yes	*DNAI1*
**3**							✓	✓	✓		Yes	
**4**							✓				No	
**5**					✓	✓					Yes	
**6**					✓	✓		✓			Yes	*CCDC40*
**7**				✓			✓				Yes	
**8**				✓			✓				Yes	
**9**							✓	✓			Yes	
**10**											No	
**11**							✓				No	
**12**				✓							No	*CFTR*
**14**							✓	✓			Yes	

**17**							✓		✓		Yes	
**19**				✓							No	
**20**							✓				No	
**21**							✓		✓		Yes	
**22**											No	
**23**											No	
**24**							✓	✓			Yes	
**25**											No	
**26**							✓				No	
**27**				✓			✓				Yes	
**28**											No	
**29**											No	
**30**							✓	✓			Yes	
**31**							✓	✓			Yes	
**32**											No	
**34**							✓	✓			Yes	
**35**											No	
**36**								✓	✓		Yes	*RSPH4A*
**37**							✓				No	
**38**							✓				No	
**39**											No	
**40**											No	

**41**							✓	✓			Yes	
**42**							✓				No	
**43**								✓	✓		Yes	
**44**											No	
**46**				✓				✓			Yes	
**48**							✓				No	
**49**									✓		No	
**50**				✓							No	
**51**							✓				No	
**52**				✓							No	
**53**											No	
**54**								✓		✓	Yes	
**55**							✓				No	
**56**					✓	✓					Yes	
**57**											No	
**58**							✓		✓		Yes	
**59**							✓	✓			Yes	*DNAH11*
**61**											No	*CFTR*
**62**							✓	✓	✓		Yes	*DNAH5*
**63**					✓	✓	✓		✓		Yes	*DNAH11*
**64**							✓		✓		Yes	*DNAH5*
**65**								✓			No	

**66**							✓	✓			Yes	
**67**							✓	✓			Yes	
**68**				✓			✓				Yes	
**69**				✓			✓	✓	✓		Yes	*DNAH11*
**Total (%)**	9 (15)	45 (74)	7 (11)								31 (51)	10 (16)

Recruitment group assigned as followed:

1) Bronchiectasis confirmed by HRCT throat with suggestive clinical features

2) Chronicsuppurative lung disease (CSLD), recurrent chest infection, recurrent otitis media +/- situs inversus and dextrocardia

3) Difficult to control asthma

Table S3: Number of SNP and Indel variants generated by rES and GS annotated and grouped by ANNOVAR

ANNOVAR Type of variant	rES	GS
**intergenic**	2573015	8475747
**intronic**	1710045	4944837
**ncRNA_intronic**	259413	828345
**UTR3**	45883	101304
**upstream**	34842	96464
**downstream**	31862	96387
**exonic**	80571	80555
**ncRNA_exonic**	22284	47386
**.**	427	23388
**UTR5**	14016	21560
**upstream;downstream**	1524	3483
**splicing**	544	627
**ncRNA_splicing**	118	285
**UTR5;UTR3**	33	53
**exonic;splicing**	28	28
**ncRNA_exonic;splicing**	8	14
**ncRNA_UTR5**	3	5
**Total**	4774616	14720468

A total of 4,774,616 and 14,720,468 SNP and Indel variants were generated via HaplotypeCaller and GenotypeGVCFs in rES and GS respectively. Since both ES and GS were capable of detecting exonic regions, ES detected 80571 exonic and 544 splicing variants, and GS was able to detect 80555 exonic and 627 splicing variants. The largest discrepancy between rES and GS in variants detected were intronic and intergenic mutations. Whilst rES primarily sequences the exonic region, library preparations are typically designed to include 50 bp from each end of each exon which would allow for the detection of intergenic and intronic variants.

Table S4: Summary statistics of back-to-back comparison of bioinformatic pipeline time taken between rES and GS (hours)

	rES			GS
	FASTQ	BAM	VCF	Full pipeline
**Time taken (hours)**	6.22 ± 3.50	3.71 ± 2.62	0.28 ± 0.11	58.05 ± 9.47

A back-to-back comparison between the bioinformatics time taken using the rES and GS pipeline was performed. After performing the initial ES, different file types could be retained for further future analysis or reanalysis if the results were negative. Typically, ES files could be kept in either FASTQ, BAM, or VCF file format. FASTQ files are raw sequence data files generated through DNA sequencers. Files kept in FASTQ format could undergo different sequence alignment algorithms for better alignment. BAM files are a compressed sequence alignment data files aligned from FASTQ files. Files kept in BAM file format could undergo new and improved variant calling algorithms to better extract true positive variants. VCF files are a text file format containing meta-information data lines with additional rows of data containing genotype information extract from BAM files. Files typically kept in VCF file format would undergo new annotations based on updated medical information about the variant. The genome sequencing full bioinformatic pipeline includes quality control, sequence alignment, and variant calling for SNP/Indels and CNVs.

Table S5: HPO Terms used for all cases by each analyst in phase 2 during analysis utilizing VarElect variant prioritization tool

Case	HPO Terms used			
	Team A – Analyst A	Team A – Analyst B	Team B – Analyst C	Team B – Analyst D
1	"primary ciliary dyskinesia", "low nasal NO", "recurrent pneumonia", "deteriorated lung function", "recurrent chest infection", "recurrent sinopulmonary infection", "microtubular disorganisation"	"primary ciliary dyskinesia"	"primary ciliary dyskinesia", "recurrent pneumonia", pulmonary, pneumonia, "recurrent sinopulmonary infection", "sinopulmonary infection", "chronic wet cough"	"primary ciliary dyskinesia", "Staphylococcus aureus", "recurrent Pneumonia", "pseudomonas", "chronic wet cough", "infectious bronchiolitis", "tuberculosis"
2	"primary ciliary dyskinesia", "outer dynein arm defect", "microtubule defect", "dyskinetic cilia"	"primary ciliary dyskinesia"	"primary ciliary dyskinesia", "allergic rhinitis", asthma, "bronchiolitis obliterans", eczema	"primary ciliary dyskinesia", lichenification, "microtubule defect"
3	"primary ciliary dyskinesia", sinusitis, "low nasal NO", "dyskinetic cilia", "microtubule defect", ADHD	"primary ciliary dyskinesia", ADHD, "low nasal NO", "sinusitis", "bronchiolitis"	"primary ciliary dyskinesia", "ADHD", "bronchiolitis", "sinusitis"	"primary ciliary dyskinesia", Bronchiolitis, Sinusitis

**5**	"biliary atresia", "primary ciliary dyskinesia", "situs inversus", dextrocardia, "respiratory distress"	"primary ciliary dyskinesia"	"primary ciliary dyskinesia", "biliary atresia", "situs inversus", dextrocardia	"Biliary atresia", "situs inversus", "Relapsing urticaria", "Bronchiectasis", "primary ciliary dyskinesia"
**7**	"primary ciliary dyskinesia", "compound cilia", epistaxis, "microtubule defect"	"primary ciliary dyskinesia", "bronchiectasis", "microtubular defect", "equinovalgus"	"primary ciliary dyskinesia“, “bronchiectasis”, “chronic wet cough”, “pneumonia”	"postinfectious chronic lung changes with bronchiectasis", "microtubular defect"
**8**	"primary ciliary dyskinesia", "recurrent chest infection", asthma, "slow beating frequency", "obstructive lung disease", "low nasal NO", "chronic lung disease"	"primary ciliary dyskinesia", "Recurrent chest infection", "asthma", "slow beating frequency", "obstructive lung disease", "low nasal NO", "chronic lung disease"	"primary ciliary dyskinesia“, “recurrent chest infection”, asthma, "allergic rhinitis", "recurrent sinusitis"	"primary ciliary dyskinesia", "chest infection", "asthma", "sinusitis", "rhinosinusitis", "hyperinflation"
**9**	"primary ciliary dyskinesia", "cilia defect", "low nasal nitric oxide", asthma, "allergic rhinitis"	"primary ciliary dyskinesia", "rhinitis", "ciliary dysfunction", "recurrent respiratory infections", "pneumonia"	"primary ciliary dyskinesia“, “allergic rhinitis”, asthma, "congested conjunctiva"	"primary ciliary dyskinesia", "asthma"

**10**	"primary ciliary dyskinesia", "mucus plug", "recurrent chest infection", "bronchus stenosis", "low nasal NO"	"primary ciliary dyskinesia", "aromatic I-amino acid decarboxylase deficiency", "oculogyric crisis", "dystonia", "developmental delay"	"primary ciliary dyskinesia", pulmonary, "common variable immune disorder", stenosis, "low nasal NO", "mucus plug"	"primary ciliary dyskinesia", "chest infection", "stenosis", "Asthma", "cystic fibrosis", "Common Variable Immunodeficiency", "Right middle lobe"
**17**	"primary ciliary dyskinesia", "panlobular emphysema", "bronchiolitis obliterans", "iron deficient anemia", "compromised lung function", "allergic rhinitis", "low nasal nitric oxide"	"primary ciliary dyskinesia", emphysema, "bronchiolitis obliterans", "iron deficiency anemia", "ciliary dysfunction"	"primary ciliary dyskinesia“, “allergic rhinitis”, asthma, "bronchiolitis obliterans", eczema	"primary ciliary dyskinesia", "bronchiolitis obliterans", "panlobular emphysematous"
**19**	IUGR, "cleft hand", "low nasal NO", "primary ciliary dyskinesia", asthma, ADHD, ectrodactyly	"primary ciliary dyskinesia", "cleft hand", asthma	"primary ciliary dyskinesia“, “mild allergic rhinitis”, asthma	asthma, "chronic wet cough", "primary ciliary dyskinesia"
**25**	"primary ciliary dyskinesia", "necrotizing pneumonia", "slow CBF", "small airway disease", pneumothorax, bronchiectasis, "low nasal NO"	"primary Ciliary dyskinesia", "bronchiectasis", "tension pneumothorax"	"primary ciliary dyskinesia“, “bronchiolitis obliterans”, “bilateral bronchiectasis”, “severe necrotizing pneumonia”, “chest infection”	"primary ciliary dyskinesia", bronchiectasis, pneumonia

**34**	"primary ciliary dyskinesia", "recurrent otitis media", "recurrent chest infection", "low nasal NO"	"primary ciliary dyskinesia", "otitis media", sinusitis, "cystic fibrosis"	"primary ciliary dyskinesia“, “recurrent chest infection”, “otitis media”, sinusitis, asthma	"primary ciliary dyskinesia", "otitis media", "chest infection", "sinusitis diagnosed"
**35**	"primary ciliary dyskinesia", "recurrent bronchiolitis", "low nasal NO", seizure	"primary ciliary dyskinesia", "otitis media", seizures, bronchiolitis	"primary ciliary dyskinesia“, wheezy, “acute bronchiolitis”	wheezy, "primary ciliary dyskinesia"
**37**	"primary ciliary dyskinesia", "chronic suppurative lung disease", "low nasal NO", "compound cilia", "dyskinetic cilia"	"primary ciliary dyskinesia", "chronic suppurative lung disease"	"primary ciliary dyskinesia“, “chronic suppurative lung disease”, “pseudomonas aeruginosa”, “recurrent chest infection”, Pansinusitis	"Chronic suppurative lung disease", "chest infection", "pansinusitis", "primary ciliary dyskinesia"
**39**	"primary ciliary dyskinesia", "low nasal NO", "recurrent chest infection", "allergic rhinitis", “microtubular defect”	"primary ciliary dyskinesia", conjunctivitis, "cystic fibrosis", "respiratory tract infections"	"primary ciliary dyskinesia“, “allergic rhinitis”, “recurrent chest infections”	"chest infection", "primary ciliary dyskinesia"
**40**	"primary ciliary dyskinesia", "necrotizing pneumonia", "microtubular defect", "chest infection", sinusitis	"primary ciliary dyskinesia", "necrotizing pneumonia", "cystic fibrosis", "respiratory tract infections"	"primary ciliary dyskinesia“, “necrotizing pneumonia”, “parapneumonic effusion”, “clinical sinusitis”, “severe chest infections”	"primary ciliary dyskinesia", Pneumonia

**41**	"primary ciliary dyskinesia", "recurrent respiratory infections", sinusitis, "acute otitis media", "very low nasal NO"	"primary ciliary dyskinesia", "recurrent respiratory infections", "cystic fibrosis"	"primary ciliary dyskinesia“, pneumonia, “Recurrent chest infection”, sinusitis, “otitis media”	"primary ciliary dyskinesia", "Asthma", "sinusitis", "acute otitis media"
**42**	"primary ciliary dyskinesia", "chest infection", "otitis media", "microtubular defect", "allergic rhinitis", asthma	"primary ciliary dyskinesia", asthma, rhinitis, respiratory	"primary ciliary dyskinesia“, asthma, “allergic rhinitis”,	Asthma, "primary ciliary dyskinesia", "Chest infection", "otitis media"
**43**	"primary ciliary dyskinesia", "congenital pneumonia", "recurrent otitis media", ADHD, "autism spectrum disorder", "chest infection"	"primary ciliary dyskinesia", "beta thalassaemia", "cystic fibrosis", "respiratory tract infections"	"primary ciliary dyskinesia“, “congenital pneumonia”, “wheezy attacks”, “chest infections”, asthma, “allergic rhinitis”	"primary ciliary dyskinesia", "chest infection", "otitis media"
**44**	"primary ciliary dyskinesia", rhinosinusitis, "low nasal NO", "fatty liver", asthma, "allergic rhinitis"	"primary ciliary dyskinesia", rhinosinusitis, "cystic fibrosis", "respiratory tract infection"	"primary ciliary dyskinesia“, asthma, rhinosinusitis, “allergic rhinitis”	Asthma, "primary ciliary dyskinesia", rhinosinusitis
**46**	"primary ciliary dyskinesia", "bronchiolitis obliterans", "small airway disease", "low nasal NO", "low IgG"	"primary ciliary dyskinesia", "bronchiolitis obliterans", "cystic fibrosis", "respiratory tract infections"	"primary ciliary dyskinesia“, “bronchiolitis obliterans”, "recurrent chest infection"	"bronchiolitis obliterans", "Otitis media", "Geographical tongue", "ciliary dyskinesia"

**48**	"primary ciliary dyskinesia", hyperphosphatasemia, "autism spectrum disorder", "allergic rhinitis", "hypopigmented lesion", "tuberous sclerosis", "hypomelanosis of ito", "low nasal NO"	"primary ciliary dyskinesia"	"primary ciliary dyskinesia“, “autism spectrum disorder”, “allergic rhinitis”	"food allergy", Eczema, "primary ciliary dyskinesia"
**50**	"primary ciliary dyskinesia", "bronchiolitis obliterans", "oropharyngeal dysphagia", "recurrent infection"	"primary ciliary dyskinesia", "bronchiolitis obliterans"	"primary ciliary dyskinesia“, “bronchiolitis obliterans”, "recurrent chest infection", wheezy	"Bronchiolitis obliterans", "chest infection", "primary ciliary dyskinesia"
**52**	"primary ciliary dyskinesia", "pneumococcal pneumonia", "hemolytic uremia", "low nasal NO", ADHD, "restrictive lung"	"primary ciliary dyskinesia", "pneumococcal pneumonia", "haemolytic uraemic syndrome"	"primary ciliary dyskinesia“, pneumonia, “haemolytic uremic syndrome”, “restrictive lung”	"primary ciliary dyskinesia", pneumonia, "haemolytic uraemic syndrome", "cardiac arrest"
**54**	"primary ciliary dyskinesia", bronchiectasis, "no cilia", clubbing, "low nasal NO", "restrictive lung", "obstructive lung", "Secretory otitis media"	"primary ciliary dyskinesia", bronchiectasis	"primary ciliary dyskinesia”, bronchiectasis, bronchiolitis	"neonatal respiratory distress", "bronchiectasis", "otitis media", "primary ciliary dyskinesia"

**55**	"primary ciliary dyskinesia", sinusitis, "recurrent pneumonia", "very low nasal NO"	"primary ciliary dyskinesia", asthma, sinusitis	"primary ciliary dyskinesia“, “recurrent pneumonia”, sinusitis	pneumonia, sinusitis, "primary ciliary dyskinesia"
**57**	"primary ciliary dyskinesia", "recurrent pneumonia", "low nasal NO"	"primary ciliary dyskinesia", asthma	"primary ciliary dyskinesia“, “recurrent pneumonia”, asthma	pneumonia, asthma, "primary ciliary dyskinesia"
**59**	"primary ciliary dyskinesia", "sinopulmonary infection", "low nasal NO", "static cilia", "wet cough", bronchiectasis, "otitis media", "recurrent pneumonia"	"primary ciliary dyskinesia"	"primary ciliary dyskinesia", “recurrent sinopulmonary infections”, pneumonia	"Sinopulmonary infection", "middle lobe syndrome", "primary ciliary dyskinesia"
**62**	"primary ciliary dyskinesia", "dynein arm defect", "microtubule defect", bronchiectasis, bronchomalacia, "congenital pneumonia", sinusitis	"primary ciliary dyskinesia", "perinatal pneumonia"	“primary ciliary dyskinesia”, Bronchiectasis, bronchomalacia, pneumonia	"primary ciliary dyskinesia", "MLS", bronchiectasis
**64**	Diagnosis achieved during the analysis of case 62	Diagnosis achieved during the analysis of case 62	Diagnosis achieved during the analysis of case 62	Diagnosis achieved during the analysis of case 62

Table S6: Summary analysis time taken each analyst in phase 2 for rES and GS

	**Type of Sequencing**	**Individual Analyst Time taken (minutes)**				**Team average analyst time taken (minutes)**	
		Analyst A	Analyst B	Analyst C	Analyst D	Team A	Team B
**Analysis time (minutes)**	rES	75.21 ± 27.01	117.07 ± 22.22	96.93 ± 55.62	88.93 ± 53.06	96.14 ± 32.40	92.93 ± 54.50
	GS	110.33 ± 53.04	123.47 ± 29.22	104.21 ± 63.42	103.71 ± 65.65	116.90 ± 43.32	103.96 ± 64.54

A back-to-back comparison between the analysis time taken in rES and GS was performed. This is because GS typically generates more variants and data which would effectively increase the analysis time. The analysis time taken for each analyst and team was recorded. The analysis time for case 7 was not taken by Team B - Analyst D. The analysis time for cases 62 and 64 were combined since they were in the same family cohort. The team average time taken for analyzing rES was 96.14 and 92.93 minutes by Team A and B respectively. This is similar to the time taken for analyzing GS at 116.90 and 103.96 minutes by Team A and B respectively. This shows that the analysis level between both teams are similar and won't impact the back-to-back comparison.

Figure S1: Coverage comparison between rES and GS across 139 Genomics England PanelApp PCD genes v1.29

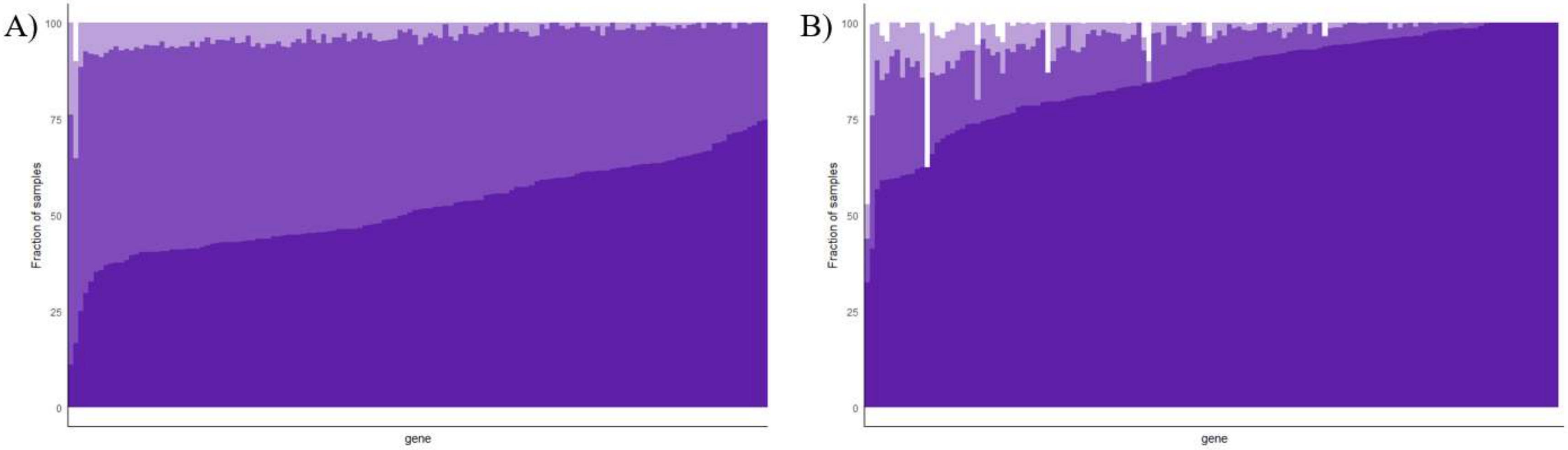

The plot showed the median read coverage of samples with at least 10× (dark purple), 20× (medium purple), and 30× (light purple) coverage over the 139 PCD genes exonic region. A – 135 genes attained a minimum of 10× coverage in 90% of samples for exome sequencing. Notable genes unable to attain at least 10× coverage includes *BBS5*, *CLRN1*, *CRX*, and *GDF1* in rES. B – A total of 138 genes attained the 10× coverage in 90% of samples for genome sequencing. Only *RPGR* did not meet the coverage requirements.
